# Acute Coronary Syndrome Presenting With Hiccups

**DOI:** 10.7759/cureus.16244

**Published:** 2021-07-07

**Authors:** Jacquelyn Hovey, Talha Perwez, Prudhvi Regula, Benjamin Chaucer, Vishnu Nagalapuram

**Affiliations:** 1 Internal Medicine, University of Alabama at Birmingham (UAB) School of Medicine, Montgomery, USA; 2 Internal Medicine, University of Alabama at Birmingham (UAB) Montgomery, Montgomery, USA

**Keywords:** hiccup, myocardial ischemia, coronary artery bypass graft, myocardial infarction, acute coronary syndrome

## Abstract

Acute coronary syndrome can present with atypical symptoms. Hiccups, generally considered benign and self-limiting, can be an indicator of myocardial ischemia if persistent. We present the case of a 62-year-old gentleman with a past medical history significant for hypertension, type II diabetes mellitus, and ischemic stroke who presented with persistent hiccups. Coronary angiogram revealed severe triple vessel disease and he underwent coronary artery bypass graft surgery, following which his hiccups resolved. There are very few cases that report the association of hiccups and myocardial ischemia. To our knowledge, this is the first reported case in which hiccups were a part of the primary symptoms associated with severe triple vessel coronary artery disease. This could be due to irritation of the phrenic nerve from the infarcted myocardium resulting in activation of the hiccup reflex arc. Our case highlights the association between these two common entities and stresses the importance of having a high index of suspicion, especially among high-risk and elderly patients.

## Introduction

Acute coronary syndrome (ACS) can manifest with atypical symptoms, especially among women and the elderly [1-3]. Hiccup, although a seemingly trivial symptom, has been rarely associated with myocardial ischemia. During these rare instances, hiccup has been a manifestation of both non-ST-segment elevation myocardial infarction (NSTEMI) and ST-segment elevation myocardial infarction often requiring percutaneous coronary intervention. To the best of our knowledge, we report the first case where hiccup was the presenting symptom for severe triple vessel coronary artery disease requiring coronary artery bypass grafting (CABG) [4-10]. Through this case, we aim to educate physicians who practice Emergency Medicine and Internal Medicine and often encounter patients with benign symptoms but could have a more serious underlying disease, especially those who are elderly and have pertinent risk factors.

## Case presentation

A 62-year-old gentleman with a history of hypertension, type II diabetes mellitus, and cerebrovascular accident presented to the emergency department (ED) with persistent hiccups for one week. He was seen by his primary care physician five days prior to the presentation for his hiccups and was given baclofen and omeprazole. He reported that his hiccups were persistent despite taking these medications, which prompted him to receive a further evaluation in the ED. He also endorsed progressively worsening shortness of breath and intermittent, burning epigastric pain for the past week.

**Figure 1:**
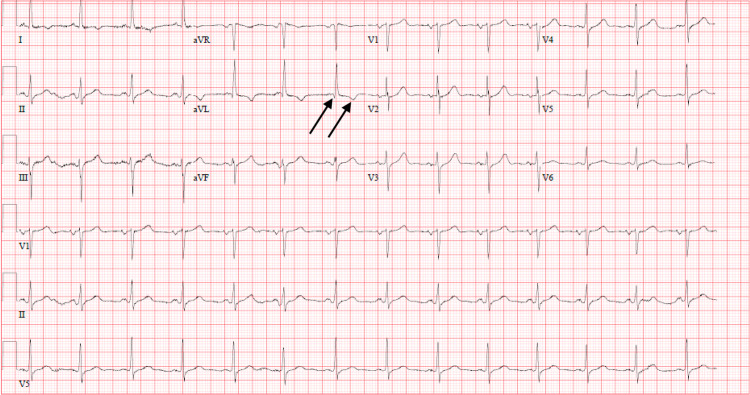
ECG on admission. Arrows denote Q-wave and T-wave inversion in lead aVL. ECG: electrocardiogram

Upon presentation, he was afebrile with a heart rate of 79 beats per minute and blood pressure of 174/104 mmHg. His physical examination was unremarkable. His chest X-ray was normal, and an electrocardiogram (ECG) revealed normal sinus rhythm with a Q-wave and T-wave inversion in lead aVL (Figure 1). His initial high-sensitivity troponin I was 2,293 ng/L, which increased to 2,436 ng/L in approximately eight hours (normal: 2.5-53.48 ng/L). Subsequently, a diagnosis of NSTEMI was established. The patient was started on aspirin, atorvastatin, carvedilol, ramipril, and therapeutic subcutaneous enoxaparin until he received cardiac catheterization the following day. For his hiccups, he was started on aluminum hydroxide and magnesium hydroxide and pantoprazole, to which he did not respond.

**Figure 2:**
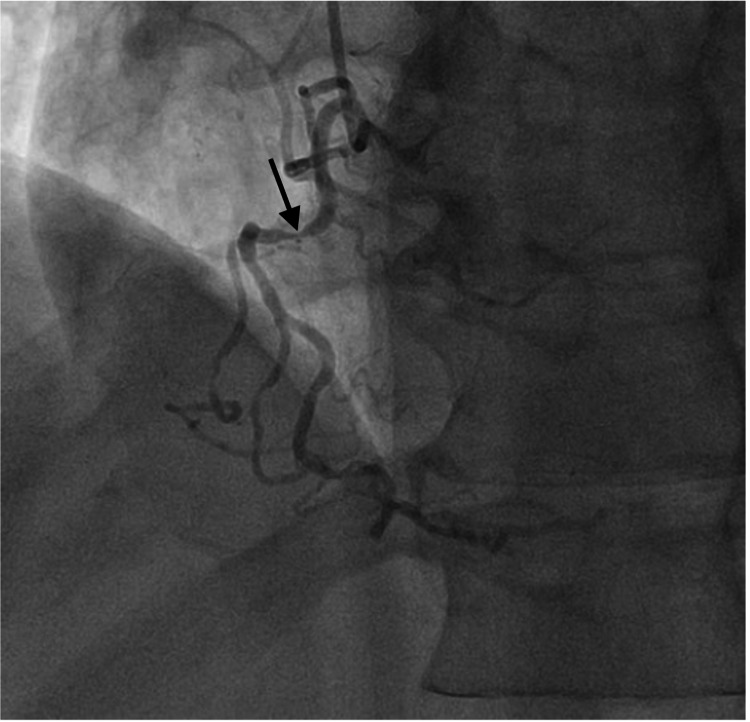
Coronary angiogram showing a 90% stenotic lesion of the proximal portion of the right coronary artery.

**Figure 3:**
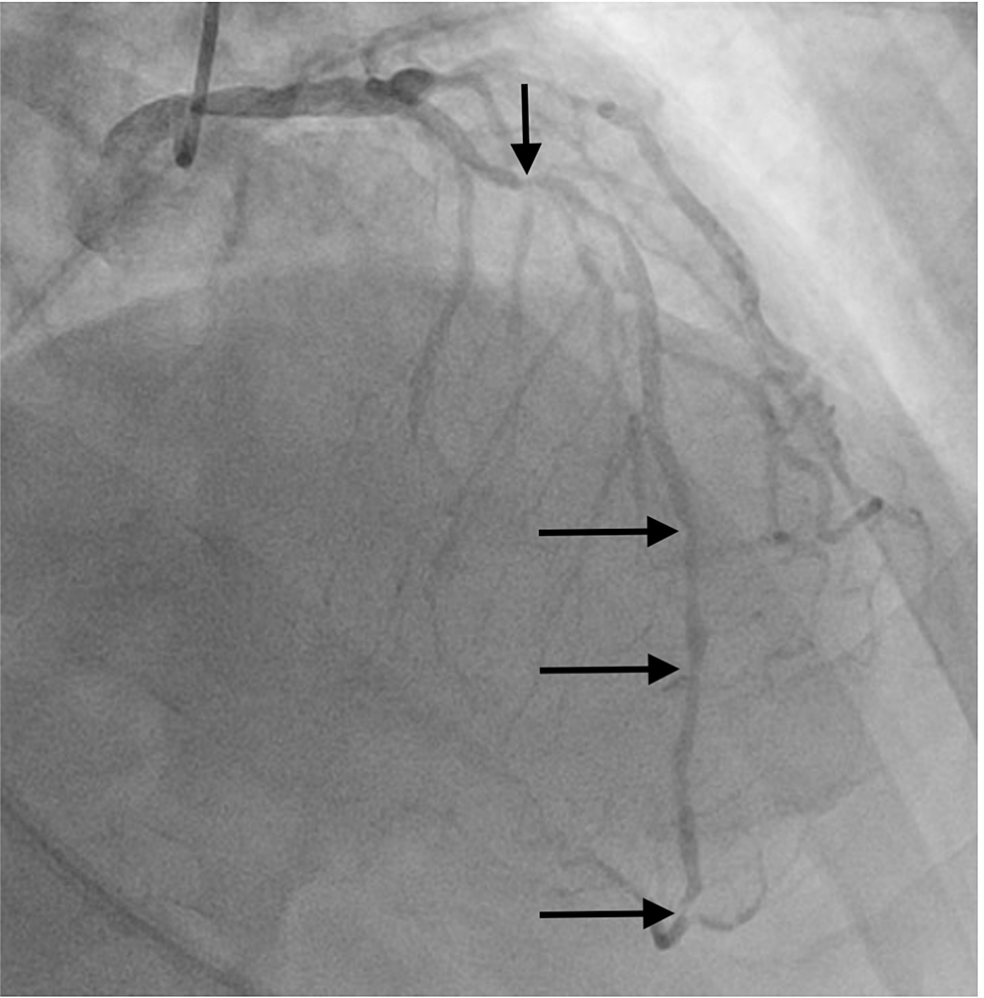
Coronary angiogram showing 80-90% stenosis of the proximal to mid-portion of the left anterior descending artery along with diffuse disease in its entirety. Prior to bifurcation into its two small terminal branches, another 90% stenotic lesion is noted.

Cardiac catheterization revealed severe triple vessel disease, including 90% stenotic lesion of the proximal portion of the right coronary artery (RCA) (Figure 2), 60-70% ostial stenosis of the left main coronary artery, 80-90% stenosis of the proximal to mid-portion of the left anterior descending artery (LAD) (Figure 3), and 80% and 90% stenotic lesions of the first and second obtuse marginal branches of the left circumflex artery (LCX), respectively, along with 90% stenosis of the remainder of the LCX itself. An early and complete revascularization strategy with CABG was determined best for this patient with severe triple vessel disease. While awaiting CABG, the patient experienced improvement of his epigastric pain and dyspnea with medical treatment but no lasting improvement of his hiccups. On the third day of his hospitalization, he underwent CABG in which three bypass grafts were implanted, namely, left internal mammary artery to LAD, saphenous vein graft (SVG) from the aorta to the acute marginal branch of the RCA, and SVG from the aorta to the first obtuse marginal artery. His postoperative period was uneventful, and he experienced immediate, lasting resolution of his hiccups following the procedure.

## Discussion

Hiccups can be associated with a wide range of etiologies and are usually self-limited and resolve spontaneously without intervention. However, in some instances, hiccups can be persistent or intractable. Such cases have been reported in association with disease or injury of the central nervous system (i.e., stroke, tumor, Parkinson’s disease, multiple sclerosis, etc.), procedures and medications (i.e., anesthetic agents, chemotherapy, azithromycin, catheter ablation, etc.), gastrointestinal disturbance (i.e., gastroesophageal reflux disease, *Helicobacter pylori*, esophageal tumor, etc.), and occasionally myocardial ischemia [11-13]. Generally, these hiccups can be managed with the treatment of the underlying cause [4-11]. Hiccup has been noted to be an atypical symptom of myocardial ischemia, especially when the RCA and inferior myocardial wall are involved [4-10]. This is thought to be due to ischemic insult to the myocardium, which activates the afferent limb of the reflex arc, which includes the vagus nerve, phrenic nerve, and sympathetic nerves. It is suspected that in such instances the phrenic nerve is irritated as it innervates the pericardium, while the vagus nerve may also become irritated as a result of myocardial ischemia of the inferior wall in particular due to its proximity [8,11,12].

Krysiak et al. reported a case where the patient was admitted for an elective surgery but had exertion-induced recurrent hiccups and retrosternal pain. Although further evaluation revealed critical lesions in LAD and RCA, hiccups resolved with stenting of the obstructed RCA alone [8]. Buyukhatipoglu et al. described two cases of chronic hiccups and atypical chest pain related to coronary artery disease, which resolved following percutaneous coronary intervention (PCI) of the culprit vessels [5]. Davenport et al. described a patient where hiccup was the sole presenting symptom of myocardial ischemia [6]. Zhang et al. reported a patient with cocaine use who presented with left-sided chest pain and underwent PCI to the mid-RCA, following which he developed hiccups related to in-stent thrombosis of the initial intervention [10]. Shaikh et al. reported a patient who was admitted with sepsis related to diabetic foot ulcer as well as hiccups. On routine evaluation, his ECG revealed ST elevation in inferior leads and was later found to have 100% mid-RCA occlusion and 80% mid-LAD lesion. Because he did not qualify for stent placement, he was managed medically. He ultimately required an implantable cardiac defibrillator for his persistently reduced ejection likely related to the above event [9]. In another interesting report, Gao et al. described a patient admitted with multiple abdominal symptoms, who eventually developed hiccups and had several ventricular arrhythmias requiring defibrillation later found to be related to an acute LAD thrombus [7]. More recently, a patient who developed hiccups during hospitalization for septic arthritis was further evaluated for ACS, given his newly diagnosed type II diabetes mellitus and hypertension, and was found to have a culprit lesion in the mid-LAD [4]. As noted from other reported cases, our patient’s hiccups, which were initially refractory to medical therapy, including baclofen, omeprazole, aluminum hydroxide, magnesium hydroxide, and pantoprazole, resolved with appropriate management of ACS.

The above cases of hiccups associated with myocardial ischemia presented in diverse ways. Either routine investigations or associated symptoms led to ACS diagnosis in most reported cases rather than etiology-directed medical decision making for hiccups alone. In our case, the patient’s age, risk factors, and associated symptoms (epigastric pain and exertional dyspnea) led us to further evaluate his persistent hiccups for myocardial ischemia. Given that ACS is often viewed as a must-not-miss diagnosis, this case stresses the importance of ACS workup in any patient presenting with persistent hiccups, especially those who are more likely to have an atypical presentation or “anginal equivalents” (dyspnea, heartburn, fatigue, and nausea/vomiting) [9,14], such as the elderly and those at high risk for ACS due to predisposing risk factors.

## Conclusions

Hiccups, though generally benign and self-limiting, may run a more protracted course. In such instances, persistent hiccups, though rare, can occasionally be indicative of a common serious pathology, such as ACS, particularly involving the RCA and inferior myocardial wall. Given significant morbidity and mortality associated with ACS, it would be prudent to further evaluate persistent hiccups for ACS, especially among elderly patients and those with pertinent risk factors. Hiccups should resolve on treating the underlying etiology, such as treatment of myocardial ischemia.
